# Medical photography: principles for orthopedics

**DOI:** 10.1186/1749-799X-9-23

**Published:** 2014-04-05

**Authors:** Metin Uzun, Murat Bülbül, Serdar Toker, Burak Beksaç, Adnan Kara

**Affiliations:** 1 Acıbadem Maslak Hospital, Darüşşafaka mah, Büyükdere cad, No. 40 Maslak, Sarıyer, İstanbul 34149, Turkey; 2 Medipol University, Istanbul 34083, Turkey; 3 Konya Meram University, Konya 42080, Turkey; 4 Acibadem University, Istanbul 34758, Turkey; 5 Şişli Etfal Education and Training Hospital, Istanbul 34156, Turkey

**Keywords:** Technique, Orthopedic, Presentation, Medical, Photograph

## Abstract

**Background:**

Medical photography is used clinically for patient evaluation, treatment decisions, and scientific documentation. Although standards for medical photography exist in many branches of medicine, we have not encountered such criteria in publications in the area of orthopedics.

**Purpose:**

This study aims to (1) assess the quality of medical images used in an orthopedic publication and (2) to propose standards for medical photography in this area.

**Methods:**

Clinical photographs were reviewed from all issues of a journal published between the years 2008 and 2012. A quality of clinical images was developed based on the criteria published for the specialties of dermatology and cosmetic surgery. All images were reviewed on the appropriateness of background, patient preparation, and technique.

**Results:**

In this study, only 44.9% of clinical images in an orthopedic publication adhered to the proposed conventions.

**Conclusions:**

Standards have not been established for medical photography in orthopedics as in other specialty areas. Our results suggest that photographic clinical information in orthopedic publications may be limited by inadequate presentation. We propose that formal conventions for clinical images should be established.

## Introduction

Medical photography is used clinically for evaluation, treatment planning, and scientific documentation. The use of clinical images enhances communication of concepts in both specialty-specific presentations and written articles. Photography was first widely used in publications particularly in the areas of dermatology and plastic and reconstructive surgeries, where the inclusion of photographic images is recognized to enhance the descriptions of diagnoses and complex procedures. In these specialties, standards for medical images have been established for publications [1-3].

Photographs that are presented in the orthopedic literature and at meetings, however, do not adhere to standard guidelines. When PubMed was searched using the terms ‘orthopedic,’ ‘medical,’ and ‘photography,’ no study was found describing guidelines for orthopedic photography. The creation of standards for photographic presentation of clinical results has enhanced the communication of ideas and information in other specialty areas. In order to investigate the consequences of the absence of standard guidelines for the use of clinical images in the orthopedic literature, this study investigated three questions:

1. Are photographic standards published for plastic surgery and dermatology appropriate for orthopedics?

2. What are applicable standards for clinical images?

3. Do recently published clinical images meet these criteria, as assessed by a survey of images in one orthopedic journal?

## Material and methods

A general orthopedics and traumatology journal was selected from those indexed in PubMed and SCI-expanded. The journal is published six times yearly. From all the articles published in the last 5 years, between 2008 and 2012, we identified and analyzed 235 clinical patient photographs. Intraoperative pictures, surgical technique diagrams, pictures that were taken in emergency departments, implant and arthroscopic camera images, and radiographs or other advanced imaging illustrations were excluded. Ethical permission was taken from the journal editorial office. Each author certifies that his or her institution has approved the reporting of this report and that all investigations were conducted in conformity with ethical principles of research.

All photographs were assessed by two observers using a modification of the published plastic surgery image guidelines according to the following criteria:

1. Background: The choice of background color should provide an appropriate contrast. How much of the image area is made up of the background?

2. Patient preparation: The extremities should be presented without clothing or accessories. There should be no visible clothing, rings, watches, or bracelets.

3. Image technique: The anatomic landmarks of the area being photographed should be visible in each image and should fill the photographed area. Using the anatomic landmarks, the subject being photographed should be in a reproducible standard position (e.g., images fully showing angle of movement of a joint: side view of knee in full flexion and extension—anterior and posterior views for varus-valgus). Thus, the representation of anatomic landmarks in each image was assessed.

**Figure 1:**
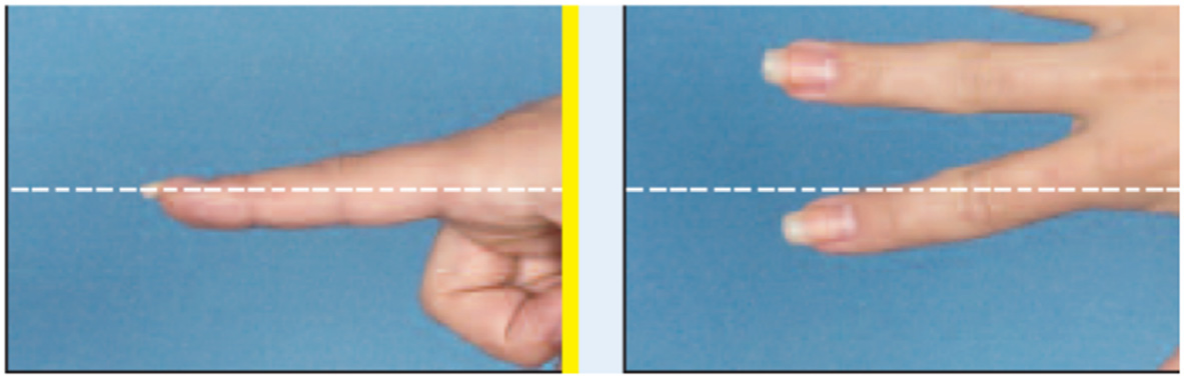
Picture showing optimal finger viewing and framing.

**Figure 2:**
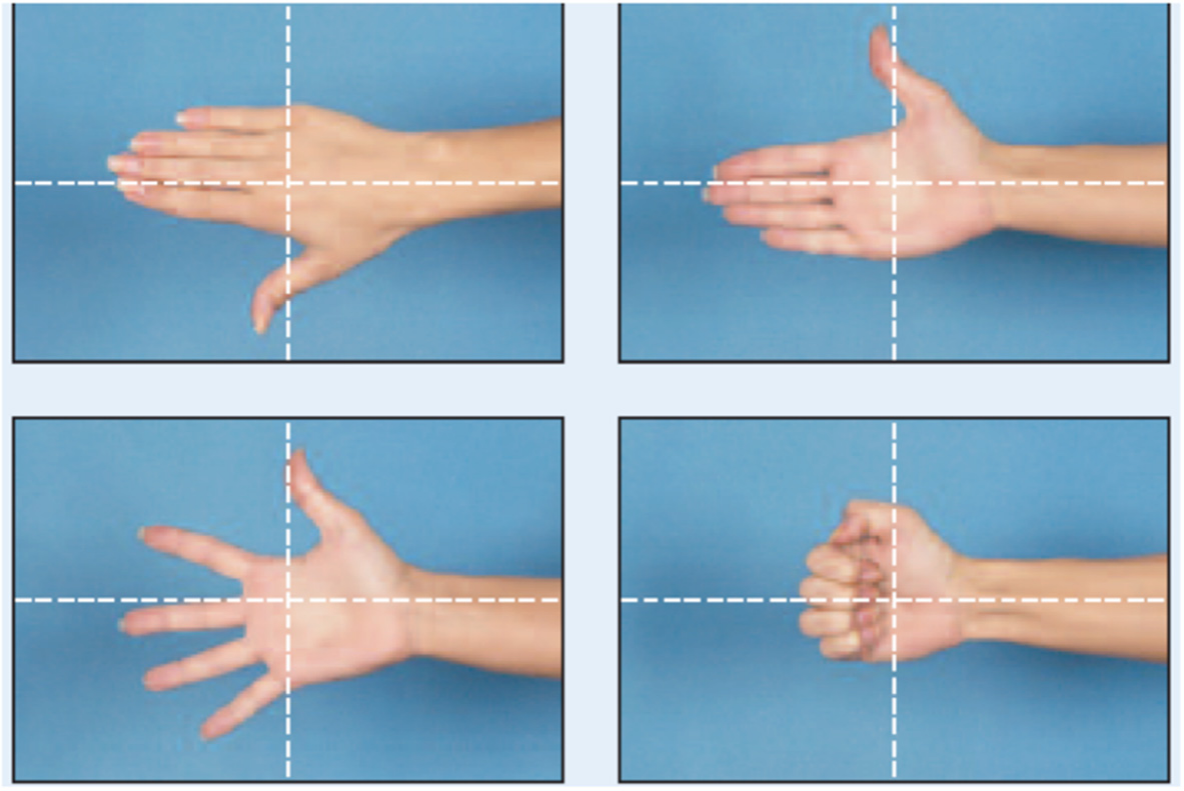
Picture showing optimal hand viewing and framing.

**Figure 3:**
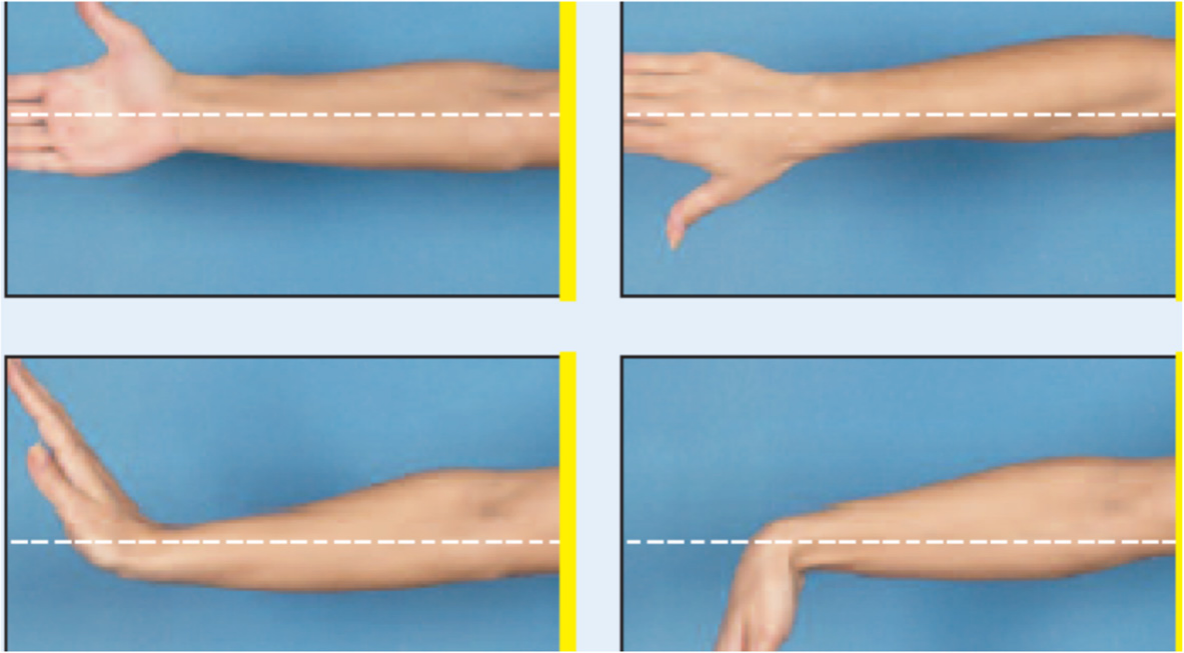
Picture showing optimal forearm and elbow viewing and framing.

Patient positioning and framing have been explained in detail for each part of the body:

1. Finger

(a) Positioning: Patient should extend the finger being examined and place it next to tape marks that are perpendicular to the camera axis.

(a) Framing: Place metacarpophalangeal joint at the edge of the frame. Center finger vertically (Figure 1).

2. Hand

(a) Positioning: Same as that of the finger.

(a) Framing: Center hand in frame (Figure 2).

3. Forearm and elbow

(a) Positioning: Patient should extend the arm and horizontally position it above the tape marks that are perpendicular to the camera axis.

(a) Framing: Place elbow at the edge of the frame and center forearm vertically (Figure 3).

4. Shoulder

(a) Positioning: Patient must comfortably stand erect with arms on the sides.

(a) Framing: Position clavicles at the top of the frame.

5. Knee and foot

(a) Positioning: Patient should stand on a step stage with foot at approximately at shoulder width.

(a) Framing: Position toes at the bottom of the frame. Center foot horizontally (Figure 4).

**Figure 4:**
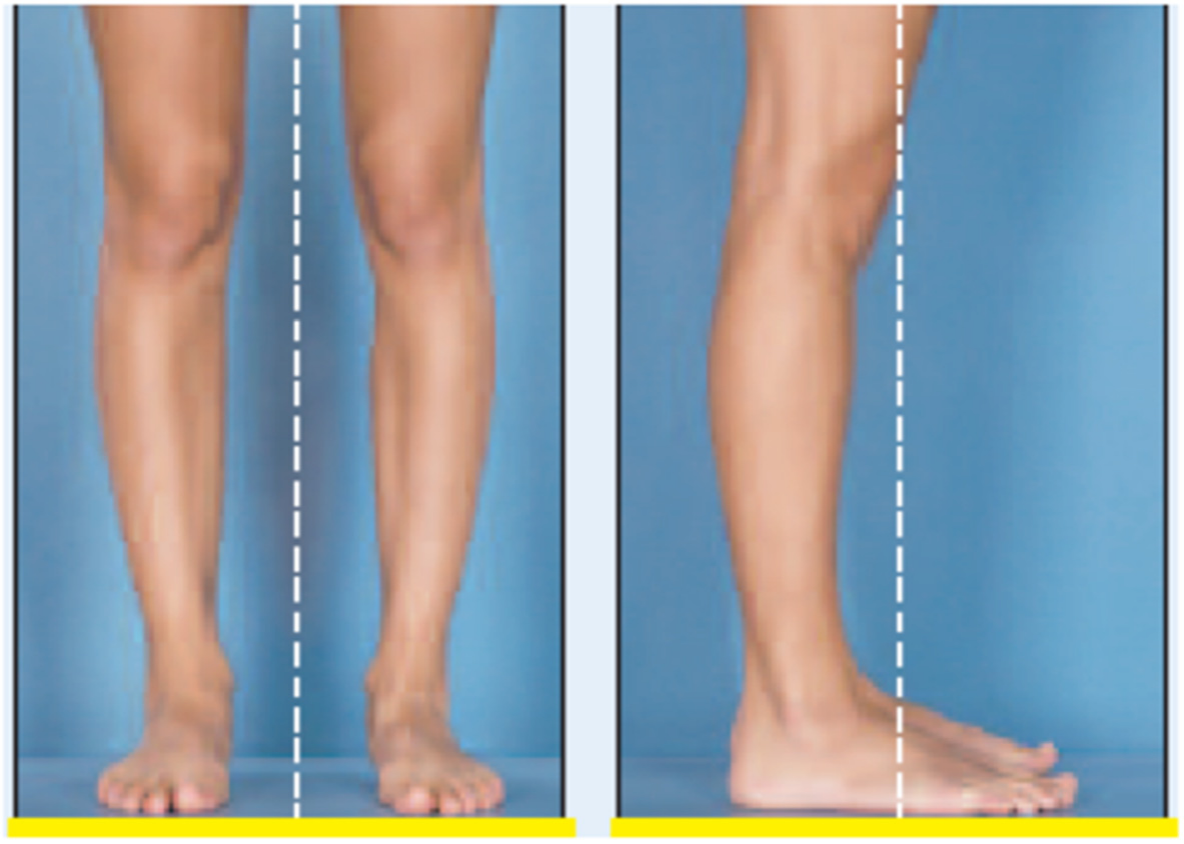
Picture showing optimal knee, leg, and foot viewing and framing.

## Results

**Figure 5:**
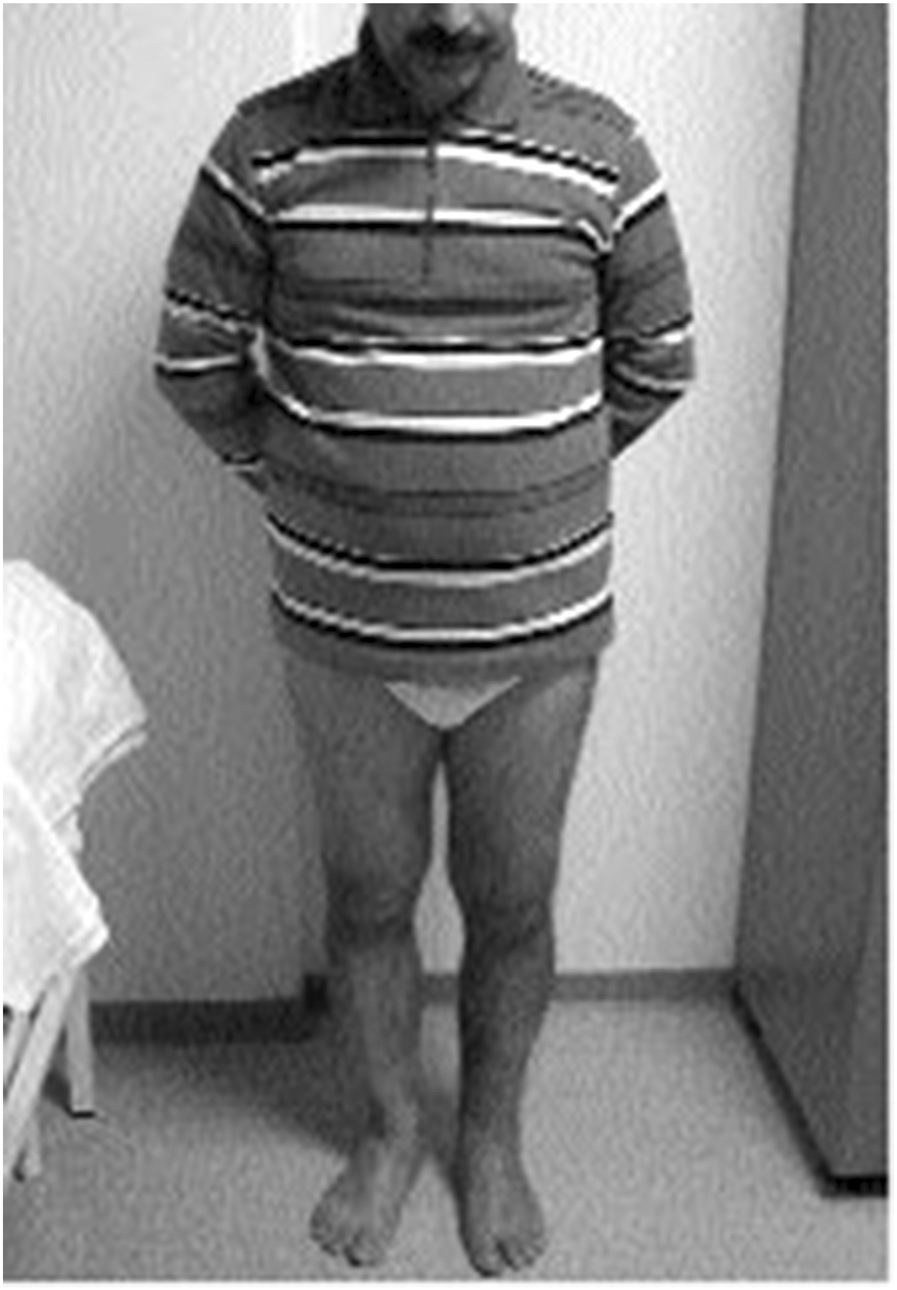
Picture showing perspective failure and without background.

**Figure 6:**
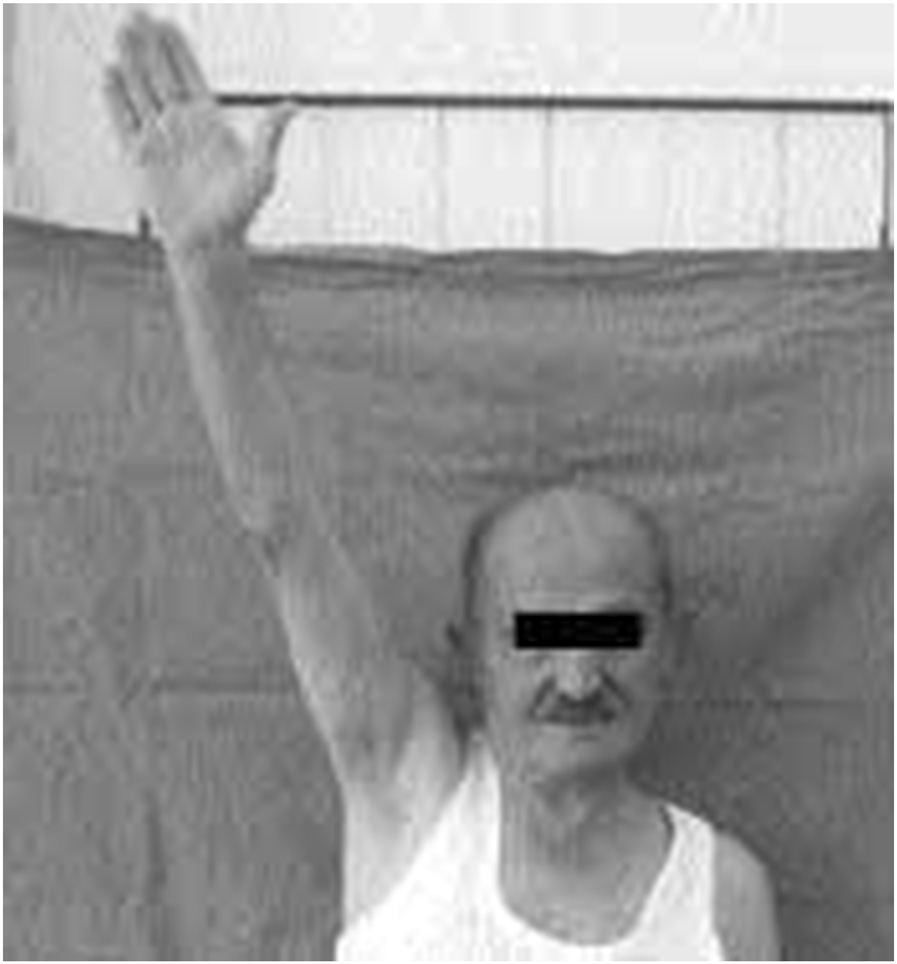
Picture showing inadequate background.

Of the 235 photographs reviewed, only 110 (44.9%) adequately met all three criteria. The other 125 (55.1%) did not meet at least one of the imaging guidelines. The reasons for inappropriacy were without background (77 photographs, Figure 5), with inadequate background (31 photographs, Figure 6), subject in wrong position (27 photographs, inappropriacy of anatomic landmarks), with errors in perspective (35 photographs, Figure 5), and visible rings, bracelets, watches, or necklaces (27 photographs). A detailed explanation of the results is given in Table 1.

**Table 1:** Inappropriate photo range

	**No background**	**Unsuitable contrast**	**Inappropriate clothing**	**Wrong position**	**Perspective failure**	**Appearance of bijou and other accessories**
Inappropriate photo number	77	108	93	27	35	27
Percentage range	57	80	69	20	26	20

## Discussion

The capture of a moment in time has always been of great interest to mankind. After many unsuccessful attempts, the moment was captured by photograph in 1816 by the French inventor, Joseph Nicephore Niepce [4]. From that date onwards, photography began to develop, and the areas of its use expanded. Photography first entered the field of medicine in 1852 when Albert Sands Southworth and Josiah Johnson Hawes recorded the first use of ether anesthesia [4]. The analog camera was launched in the market in 1888 by Kodak (Rochester, NY, USA) [4-6]. The use of this camera was limited: it was difficult to use, images could not be viewed immediately, and printing took time and was not cost effective [7]. After many advancements in technology, however, including the development of digital cameras, camera usage has become widespread in medicine, particularly in dermatology, plastic surgery, forensic medicine, anatomy, pathology, and orthopedics [1,8,9]. In plastic surgery and dermatology, photographic communication of specialty-specific information has been recognized as highly important, and the importance of standardization and quality of clinical images has been recognized [1,3,7,10-16]. These specialties therefore established standards for published clinical images to maximize the information that can be communicated by these figures.

The purpose of this study was to assess the presentable quality of images used in an orthopedic publication and to propose standards for medical photography in orthopedic surgery. No published standards exist for the presentation of orthopedic clinical images. The journal selected for this study is accepted as an international general orthopedics journal and includes a wide range of orthopedic images. Only clinical photographs, except those in the emergency or operating rooms, were evaluated in this study. Separate conventions would be useful for standardizing the presentation of radiographs, arthroscopic images, and other imaging information, but these issues are beyond the scope of this study.

The applicable accepted conventions for clinical photography in dermatology and plastic surgery describe equipment, background selection, accessories, patient preparation, and photographic techniques [2,3,17-19]. Equipment quality is important, and it has been reported that photographs should be taken with cameras that have at least 3.2 megapixels [20]. In this study, the camera features could not be evaluated, and it should be noted that photo quality may also be affected by the file format, such as TIFF, JPEG, or BIT.

In the evaluation of accessories, it has been reported that the most important accessory is a tripod to enable precise control of the camera angle [2,3,21-23]. In this study, we could not evaluate the use of a tripod, photography practices, or camera features, since the articles did not include this information.

**Figure 7:**
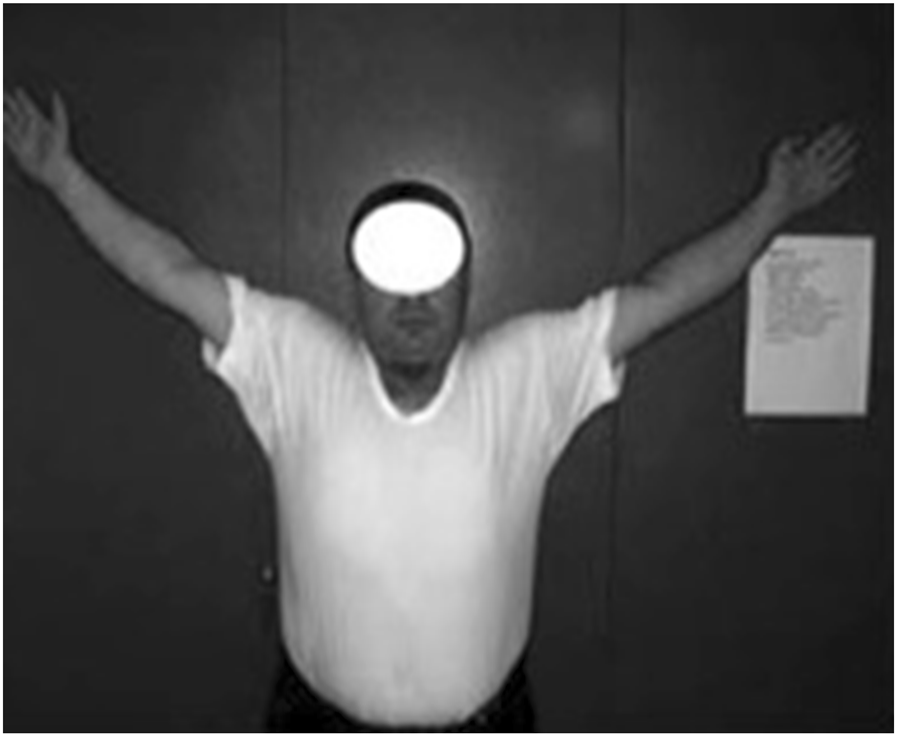
Picture showing inappropriate background.

The importance of an appropriate background is indisputable in medical photography. Background color should be chosen to provide an appropriate contrast, and guidelines exist for the relative proportions of the image that should be occupied by the subject of the photograph versus the background [2,3,24]. Dibernardo [2] and Dibernardo et al. [3] have reported that a sky blue background is preferable because other colors may intermix. Photograph of patients against a solid-colored background is preferable. Light to medium blue is a good choice because it contrasts well with skin tones. Medium gray may also work well. In our study, we did not assess the background color, just because most of the study pictures were in black and white. Another important feature of the background is that it should be uniform, and the background should not include patterns or distracting features such as tiles, furniture, electric cables, or doors [3,25] (Figure 7). When an inappropriate background was noted in the images in this study, it was observed that generally, images had been taken with the patient against a wall or on a stretcher. In the background of these images were electric sockets, computers, clocks, tables, lamps, floor tiles, waste baskets, and the feet of either the patient or the photographer. In fact, a special background had been used in only eight photographs. In addition, we frequently observed that orthopedic images also failed to frame the subject correctly, with portions of the extremity often going out of the frame as the patient performed the movement to be shown.

**Figure 8:**
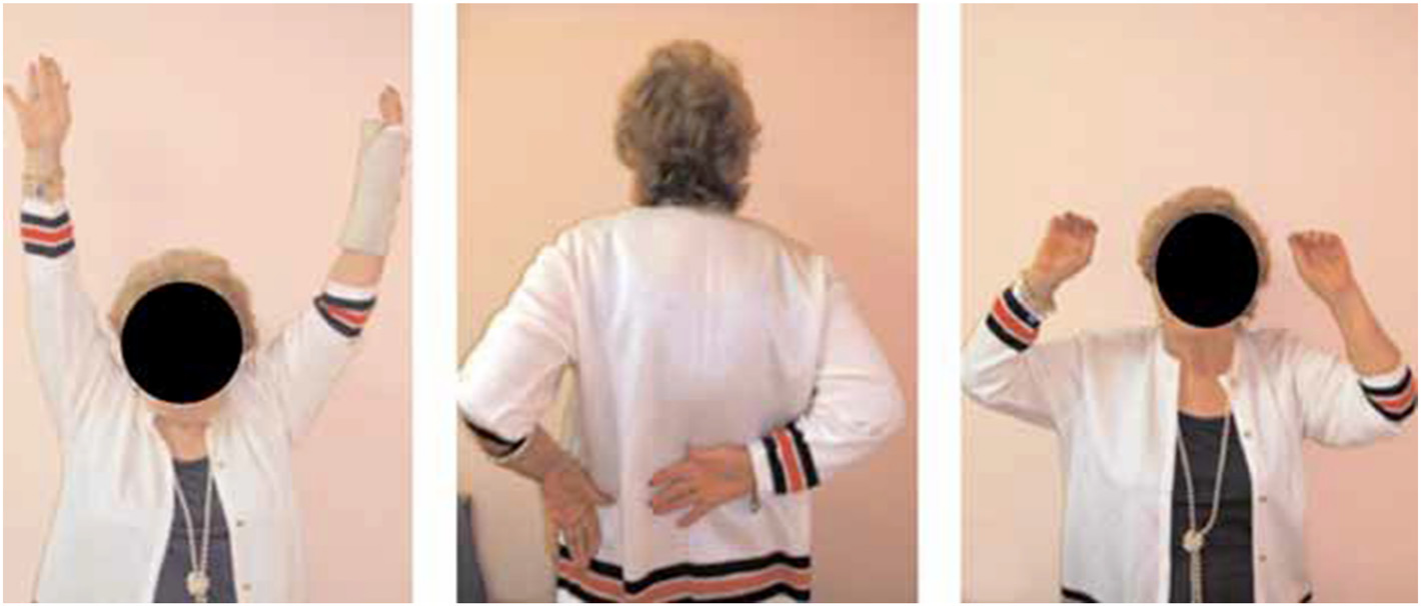
Picture showing inadequate patient preparation.

Regarding patient preparation, for both the upper and lower extremities, it has been recommended that clothing and accessories be removed from the area of interest [26,27] (Figure 8). The presence of clothing, rings, watches, or bracelets in the image may conceal anatomic features or distract focus from the subject of the photograph. This study found that 20% of clinical images included such accessories, which, with minimal effort, could have been removed prior to obtaining the image.

**Figure 9:**
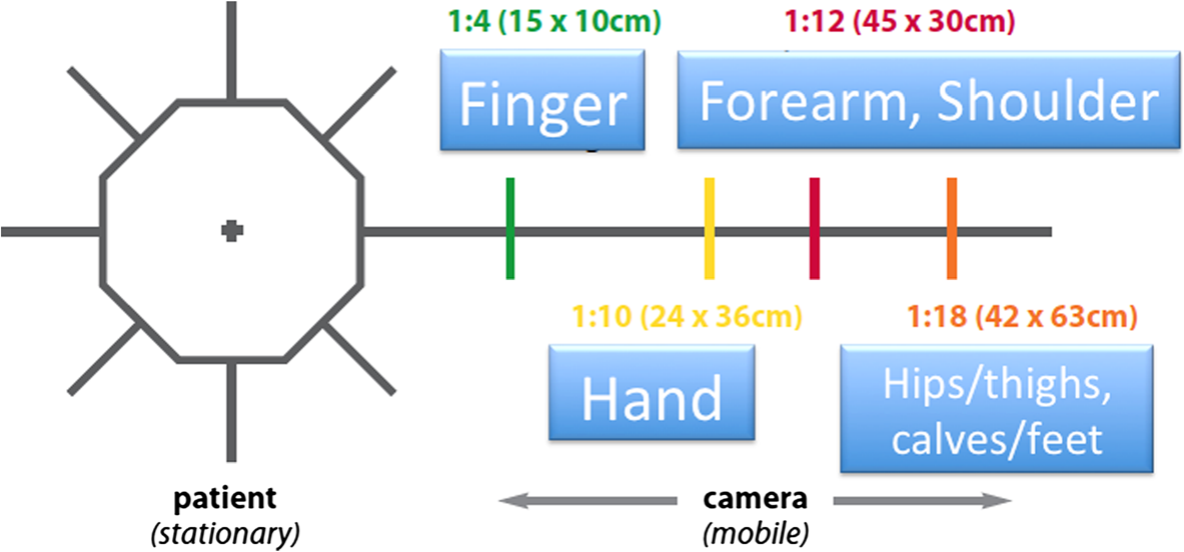
Diagram showing the distances between patient and camera.

In the evaluation of technique, the anatomic landmarks for the area being photographed should be visible in each image and should fill the photographed area. The images are marked with gridlines to assist in proper framing. When an image is meant to be framed by positioning an anatomical landmark in the center of the frame, this is indicated by a dotted line on the image (Figures 1, 2, 3 and 4). This error was detected in 20% of the images evaluated. Assuming that the clinician obtaining the image is familiar with the anatomy relevant to the clinical situation, improving this aspect requires only awareness of the need for conventions which enhance the information communicated by each image and consideration of the standard presentation of anatomy. The diagram in Figure 9 shows an overhead view of a suitable tape mark pattern. A 30-cm octagon with radiating lines is used for positioning the patient. One line is extended out along the camera axis and marked at appropriate distances. The diagram shows the distances between the camera and a part of body that is pictured. In addition to determining the appropriate orientation of the camera, the lens should be at the same level as the area to be photographed [1,3]. Errors in perspective were frequently observed in this study, particularly for images of the feet (Figure 6). When images are obtained either with the patient standing or lying on an examination bed, the camera perspective may not capture the correct anatomic proportions. Of the photographs examined, 14.3% were determined as having errors of perspective. All of the images with errors of perspective were also determined to have background mistakes.

In conclusion, this study found that clinical images in an orthopedic journal did not adhere to standards of image quality which have been previously established in the fields of dermatology and plastic surgery. Subjectively, during the review of these images, it was the opinion of the author that some images nonetheless adequately presented clinical information, but some could have been improved by adherence to the standards proposed above. In the development of the imaging criteria used in this study, an effort was made to apply only basic photographic standards appropriate for orthopedic information: background, patient preparation, and perspective technique.

Photography has become increasingly important in medicine for the communication of complex, specialty-specific clinical information. As has been established already in other specialties, in orthopedics, results should be presented using appropriate techniques (Figure 9). Standards for orthopedic images should be established to include conventions for the use of background, patient preparation, and perspective techniques. By establishing such a *lingua franca*, the quality of clinical images available in the literature would be improved, as would even an individual clinician's ability to compare pre- and post-treatment outcomes. The results of this study highlight the need for established standards for medical photography in orthopedics.

## Competing interests

Each author certifies that he or she has no commercial associations (e.g., consultancies, stock ownership, equity interest, patent/licensing arrangements, etc.) that might pose a conflict of interest in connection with the submitted article.

## Authors’ contributions

MU and MB carried out the studies, participated in the sequence alignment, and drafted the manuscript. ST carried out the assessment. AK participated in the sequence alignment, conceived of the study, participated in its design and coordination, and helped draft the manuscript. ST and BB participated in the design of the study and performed the analysis. All authors read and approved the final manuscript.
